# Safe spontaneous breathing with helmet noninvasive ventilation in acute hypoxemic respiratory failure

**DOI:** 10.1097/MCC.0000000000001345

**Published:** 2025-11-19

**Authors:** Tommaso Rosà, Luca S. Menga, Bruno L. Ferreyro, Domenico Luca Grieco, Massimo Antonelli

**Affiliations:** a Department of Emergency, Intensive Care Medicine and Anesthesia, Fondazione Policlinico Universitario A. Gemelli IRCCS; b Department of Anesthesiology and Intensive Care Medicine, Catholic University of The Sacred Heart, Rome, Italy; c Keenan Research Centre, Li Ka Shing Knowledge Institute, St Michael's Hospital, Unity Health Toronto; d Interdepartmental Division of Critical Care Medicine; e Interdepartmental Division of Critical Care Medicine, Sinai Health System/University Health Network, University of Toronto, Toronto, Canada

**Keywords:** acute hypoxemic respiratory failure, acute respiratory distress syndrome, helmet noninvasive ventilation, inspiratory effort, patient self-inflicted lung injury

## Abstract

**Purpose of review:**

Helmet noninvasive ventilation (NIV) has gained attention for the management of hypoxemic patients, owing to physiological and potential clinical benefits. We summarize the recent advances on the topic.

**Recent findings:**

Compared to facemasks, helmets facilitate application of higher positive end-expiratory pressure (PEEP) for prolonged treatments: this improves oxygenation and may mitigate injurious inflation patterns related to lung heterogeneity. The large, highly compliant interface reduces ventilator triggering performance, causing pressure support to be partially out of phase with patient's inspiratory effort; however, it allows patients to breathe from the internal air reservoir, resulting in formally asynchronous breaths that may help attenuate surges in lung stress and tidal volume without causing flow starvation. Through physiological monitoring, ventilator settings can be individualized to modulate inspiratory effort while limiting increases in dynamic transpulmonary driving pressure and tidal volume.

**Summary:**

Helmet NIV may offer a valuable strategy for noninvasive management of hypoxemic patients, particularly when applied early, for prolonged periods, and with settings aimed at minimizing injurious inflation in moderate-to-severe (PaO_2_/FiO_2_ < 200 mmHg) cases. Interface peculiarities affecting patient-ventilator interaction may constitute key differences with facemask NIV for prevention of injurious inflation patterns. Ongoing trials will clarify whether these physiological advantages improve clinical outcomes.

## INTRODUCTION

Helmet noninvasive ventilation (NIV) has emerged as a promising strategy for the initial management of moderate-to-severe acute hypoxemic respiratory failure [1,2]. Its unique design allows for the safe application of higher positive end-expiratory pressure (PEEP) and prolonged treatment sessions with improved comfort and tolerance as compared to facemask NIV [1]. Physiological evidence and early clinical trials suggest that, when applied in selected patients in the early phase of acute hypoxemic respiratory failure, helmet NIV can mitigate inspiratory effort, improve oxygenation, and potentially prevent patient self-inflicted lung injury (P-SILI), which translates into improved clinical outcomes [3^▪▪^,^4^,^5^,^6^^▪▪^]. However, the distinctive mechanical characteristics of the interface demand specific ventilator settings, attentive monitoring, and careful patient selection to optimize efficacy and safety. In this review, we discuss the use of helmet NIV, with a focus on recent evidence supporting its physiological rationale and its potential to enhance lung protection in hypoxemic patients during noninvasive respiratory support in the ICU setting. 

**Box 1:**
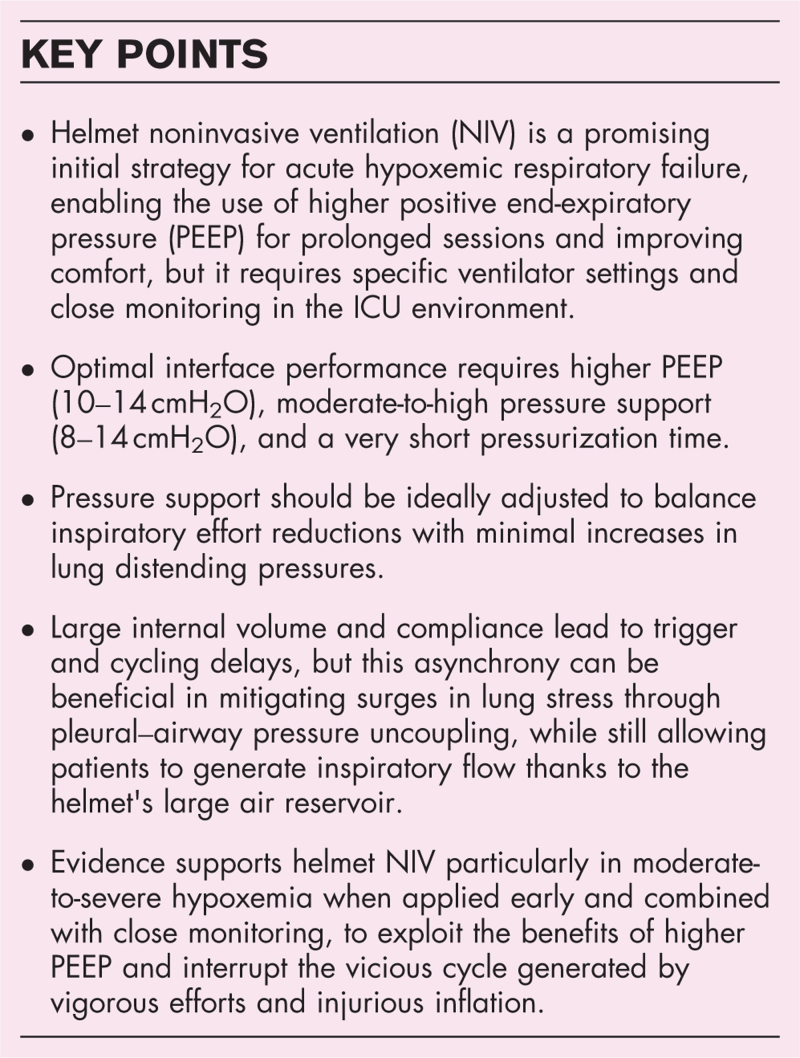
no caption available

## HELMET NONINVASIVE VENTILATION: WHAT

**FIGURE 1:**
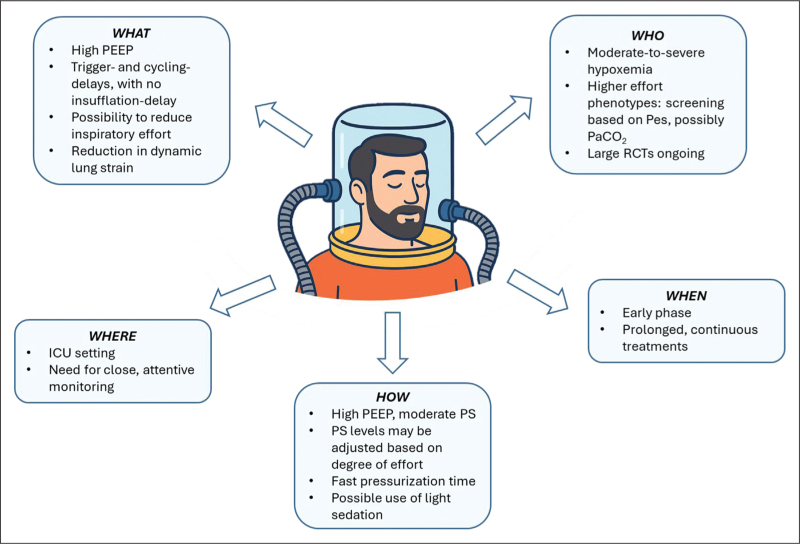
Helmet NIV: summary of indications.

Helmets can be used to deliver continuous positive airway pressure (CPAP) or pressure support ventilation (PSV) through a transparent, soft, and latex-free hood that encloses the patient's entire head, sealed around the neck by a soft collar (Fig. 1). Compared to facemasks, the helmets offer greater comfort and tolerance, allowing for longer continuous treatments without the discomfort and skin lesions frequently associated with the former [1,7–10].

The present review focuses on the application of helmet noninvasive ventilation in pressure support mode (helmet NIV). The physiological rationale for the application of helmet NIV arises from specific features of the interface that influence patient-ventilator interaction. First, helmets allow the delivery of higher levels of PEEP in comparison to facial masks safely and effectively [5,11]. Higher PEEP has the potential to mitigate the risk of P-SILI in more severely hypoxemic patients (ratio of arterial oxygen partial pressure to inspired oxygen fraction ≤200 mmHg), particularly in the early-phase of the disease [12,13^▪^]: this is achieved thanks to lung recruitment, alveolar stabilization reducing inflation heterogeneity, improved oxygenation, caudal diaphragm displacement and reduced inspiratory effort. The helmet's design distributes pressure evenly around the neck and head, minimizing discomfort, side effects like pressure-sores and air-leaks, thereby allowing higher PEEP to be applied for prolonged periods [1,5]. Earplugs may be used to attenuate the discomfort caused by the noise and pressure [14].

**FIGURE 2:**
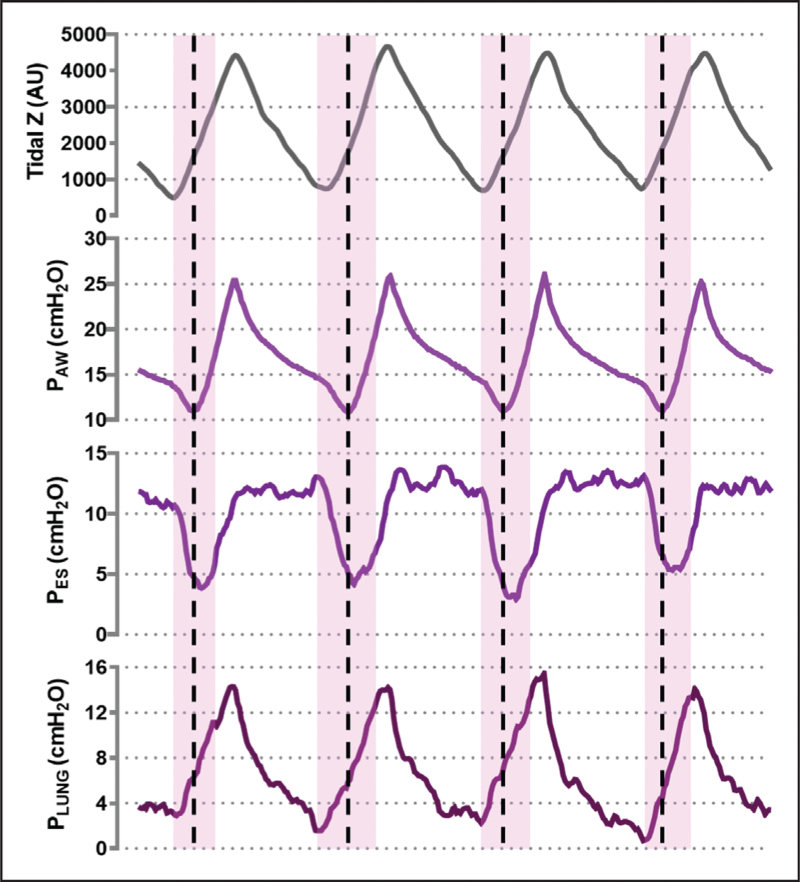
Patient-ventilator (a)synchrony. Tidal volume (tidal Z, assessed by electrical impedance tomography in arbitrary units, AU), airway pressure (*P*_aw_), esophageal pressure (*P*_es_), and transpulmonary pressure (*P*_lung_) tracings during helmet noninvasive ventilation are shown. Light pink bars indicate the phase of muscular inspiration, beginning with the initial Pes deflection and ending when Pes returns to one-third of its baseline value. Dotted lines mark the ventilator trigger and the onset of pressure support delivery, which occurs with a substantial delay. This delay represents a formal asynchrony, but it is not accompanied by a true mismatch, since muscular inspiration and flow generation start simultaneously, as seen in the tidal volume tracing. Finally, the *P*_aw_ tracings illustrate the slow rise and decay of pressure during inspiration and expiration, a phenomenon related to the high compliance of the helmet interface. The combination of a ‘formal’ asynchrony with the slow rise in *P*_aw_ helps prevent abrupt increases in transpulmonary driving pressure (*P*_aw_ – *P*_es_), thereby mitigating lung stress.

Nevertheless, the use of helmets poses specific challenges due to the increased compliance and dead space, which influence patient-ventilator interaction and require specific settings to function effectively [15–17]. These interface-related factors can contribute to delays in ventilator triggering and cycling, leading to an overall shorter time of synchrony. During helmet NIV, triggering and expiratory-cycling delays occur due to the large internal volume of the helmet and the resulting buffering of pressure changes, further enhanced by the inherent compliance of the interface. The latter also slows down the rise and decay in airway pressure throughout the respiratory cycle. To minimize these effects and enhance interface performance, higher PEEP and pressure support (PS) levels are typically utilized, together with a very short pressurization time (ramp). Of note, however, the remaining degree of asynchrony does not necessarily represent an undesirable occurrence, and may even be beneficial. Importantly, during asynchronous triggering and cycling-off, when ventilator valves are closed, patients can still generate airflow from or exhale in the interface's internal volume (Fig. 2). This prevents isometric contractions and the sensation of flow starvation. Unlike facemask NIV, trigger delays do not translate into delayed or prolonged lung inflation. Intense isometric contractions (i.e. without flow generation) occurring during the trigger phase may contribute to increasing discomfort and inspiratory effort, and, importantly, promote the occurrence of local heterogeneities (i.e. pendelluft), leading to local overstretch in dependent lung regions [18,19]. However, during helmet NIV, the possibility to generate flow from the internal volume before inspiratory valve opening helps prevent the aforementioned phenomenon. In addition, the uncoupling of negative pleural pressure swings from ventilator-generated airway pressure surges helps mitigate breath-by-breath increases in transpulmonary driving pressure (lung stress) that would be caused by full synchronization [20,21]. The combined effects of high PEEP improving oxygenation and lung recruitment, pressure support unloading the respiratory muscles, and a moderate degree of asynchrony mitigating rises in transpulmonary driving pressure collectively contribute to the reduction in inspiratory effort and dynamic lung strain observed with helmet NIV as compared to high-flow nasal oxygen [3^▪▪^], although tidal volume may be increased [3^▪▪^,^4^]. Compared to helmet CPAP, helmet NIV primarily reduces inspiratory effort [3^▪▪^,^4^,^22^].

## HELMET NONINVASIVE VENTILATION: WHO

Guidelines do not provide conclusive recommendations for or against the use of helmet NIV in hypoxemic patients [23^▪▪^,^24^,^25^]. Nevertheless, NIV is frequently used in this setting with variable success rates, and relevant concerns exist regarding the potential of facemask NIV to delay intubation and increase mortality [26,27]. Recently, the clinical efficacy of helmet NIV in moderate-to-severe acute hypoxemic respiratory failure has been suggested in large meta-analyses [2,28,29] and increasingly supported by a few randomized controlled trials (RCTs) conducted in this patient population, which indicate that helmet NIV may outperform other noninvasive strategies [5,6^▪▪^]. However, the evidence is still limited to specific settings and samples (recently mostly restricted to COVID-19 ARDS) and stems primarily from single center trials [5] or from studies conducted in centers with long lasting experience in its use [6^▪▪^], hampering any conclusive evaluation of the clinical benefit, if any. In this sense, the largest RCT conducted in patients affected by COVID-19, showed no differences in clinical outcomes between helmet NIV and a control group treated with a combination of other noninvasive strategies [30^▪▪^]. The patients enrolled in this trial had severe hypoxemia and were included at a relatively advanced stage in the progression of respiratory failure. This raises the concern that they may have been recruited outside the optimal clinical window during which the physiological benefits of helmet NIV – such as the prevention of further lung injury via P-SILI – could be realized. Additionally, a significant limitation of the study was the lack of standardization in the control group, which saw high rates of NIV use as well. Taken together, these studies suggest that helmet NIV is a very promising first-line strategy for acute hypoxemic respiratory failure, especially when initiated early and applied for prolonged periods in cases of moderate-to-severe hypoxemia (PaO_2_/FiO_2_ < 200 mmHg) [31]. However, similar to all other noninvasive respiratory support strategies, escalation to invasive ventilation should not be delayed when noninvasive treatment is failing [27,32]. With this regard, the helmet poses significant monitoring challenges as compared to facemask NIV, as tidal volume and P0.1 cannot be reliably assessed at the bedside. Moreover, even with optimized settings, helmet support strategies are associated with minimal – but still present – amount of CO_2_ rebreathing, which must be carefully evaluated through arterial blood gas measurements [33,34]. Larger, multicenter RCTs comparing helmet NIV, HFNO, and helmet CPAP are currently underway (ID: NCT05089695, ID: NCT05078034).

**FIGURE 3:**
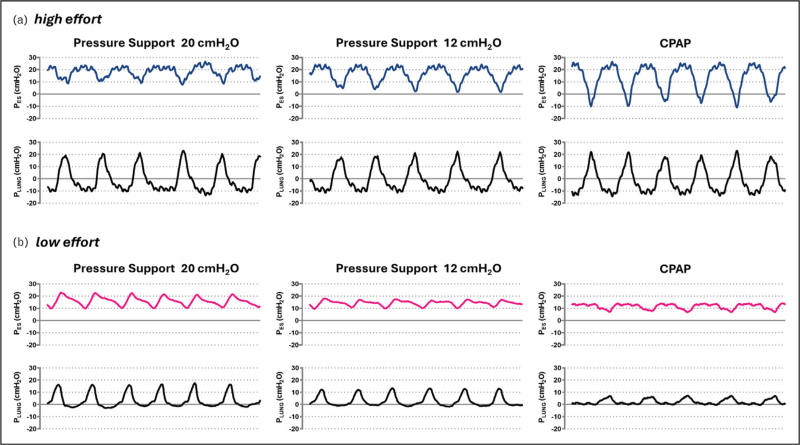
Esophageal and transpulmonary pressure tracings in two hypoxemic patients during helmet NIV at different support levels and helmet CPAP. Panel (a) illustrates the mechanics of a patient with very intense inspiratory effort, evidenced by large negative swings in esophageal pressure. In this case, increasing pressure support effectively mitigates muscular effort: the marked reduction in esophageal (pleural) pressure swings counterbalances the additional pressure applied, thereby maintaining dynamic transpulmonary driving pressure constant. A pressure support of 20 cmH_2_O is thus associated with improved spontaneous breathing mechanics. Conversely, Panel (b) depicts a patient with lower inspiratory effort, where rising levels of pressure support lead to a gradual increase in dynamic transpulmonary driving pressure. Here, over-assistance is evident from the esophageal pressure tracing, which shows an abnormal upswing during inspiration due to excessive airway pressurization. In this setting, helmet CPAP provides the most balanced strategy, simultaneously limiting lung stress and avoiding undue suppression of effort. CPAP, continuous positive airway pressure; NIV, noninvasive ventilation.

Meanwhile, available physiological evidence highlights how helmet NIV may be especially beneficial in selected patient subgroups. In a posthoc analysis of the HENIVOT trial comparing helmet NIV and HFNO [6^▪▪^], lower baseline arterial carbon dioxide pressure (PaCO_2_ < 35 mmHg) identified patients with a higher likelihood of benefiting from helmet NIV in terms of avoidance of endotracheal intubation [35]. When esophageal monitoring is not feasible, PaCO_2_ can serve as a surrogate marker for higher minute ventilation resulting from more intense efforts, aiding clinicians in the identification of distinct patient phenotypes or in the evaluation of disease severity. Similarly, two small physiological randomized-crossover trials comparing helmet NIV with HFNO and helmet CPAP reported greater reductions in inspiratory effort with helmet NIV, particularly in patients exhibiting high effort during HFNO [3^▪▪^,^4^]. In these patients, effort reduction offsets the increase in airway pressure caused by pressure support, finally resulting in unchanged, or even lower, transpulmonary driving pressure. Conversely, patients with moderately low inspiratory effort during HFNO experienced an increase in driving transpulmonary pressure with helmet NIV compared to helmet CPAP, despite similar improvements in oxygenation. These findings suggest that pressure support levels during helmet NIV should be individualized to control inspiratory effort while minimizing increases in driving transpulmonary pressure (lung stress). Helmet NIV (with variable pressure support) appears most suitable for patients with high inspiratory effort, as HFNO and helmet CPAP are insufficient to control it [3^▪▪^,^22^]. In contrast, helmet CPAP and HFNO may be preferable in hypoxemic patients with milder effort, offering oxygenation benefits without increasing lung stress (Fig. 3). However, for such a personalized physiology-guided approach, accurate effort assessment is necessary. Research is ongoing to identify practical techniques for accurately assessing inspiratory effort in nonintubated patients, which remains challenging in routine clinical practice beyond research settings [36^▪▪^,37^▪^].

Taken together, most recent evidence indicates that helmet NIV may yield the most physiological and clinical benefits in moderate-to-severe hypoxemic patients (PaO_2_/FiO_2_ < 200 mmHg), especially when they exhibit intense inspiratory effort (>10 cmH_2_O).

## HELMET NONINVASIVE VENTILATION: WHEN

The timing of helmet NIV initiation can critically influence its efficacy in acute hypoxemic respiratory failure. Early application of high PEEP during the initial, more acute phase – when response to PEEP is often maximal – may provide the greatest benefit [38]. Alveolar recruitment, combined with reduced inspiratory effort, may lower lung stress and dynamic lung strain, thereby attenuating the inflammatory cascade and slowing the progression of injury [3^▪▪^,13^▪^]. Indeed, strong inspiratory efforts, which markedly increase lung stress and promote injurious phenomena such as pendelluft, may exacerbate lung injury if undetected and sustained over time [19,39]. Differences in outcomes observed in trials initiating treatment early versus later with respect to symptoms onset or ICU admission seem to confirm this physiologically sound hypothesis [6^▪▪^,^30^^▪▪^]. In contrast, delayed initiation often coincides with lung injury progression toward more consolidated, less recruitable tissue, where the benefits of PEEP on strain reduction are limited.

Prolonged, continuous helmet NIV sessions appear advisable to maximize effectiveness until clinical improvement occurs. If interruptions are required – due to intolerance or feeding needs – a temporary switch to HFNO may be reasonable. Albeit such breaks can precipitate de–recruitment and worsen oxygenation, they may also serve as helmet–weaning trials, providing insight into disease trajectory and readiness for support reduction. One potential, yet undemonstrated, benefit of helmet compared to facemask NIV could be that interruptions for intolerance and discomfort are seldom necessary.

## HELMET NONINVASIVE VENTILATION: WHERE

In the noninvasive management of severely hypoxemic patients, clinicians must carefully balance the risks associated with intubation and sedation for invasive mechanical ventilation [40] against those of delayed intubation and P–SILI [39]. This is a particularly challenging task in which careful monitoring plays a pivotal role. Importantly, all trials suggesting a clinical benefit of helmet NIV have been conducted in intensive care units [5,6^▪▪^]. It is likely that the improved outcomes are due not to the technique itself, but to the combination of helmet NIV with comprehensive critical care support and monitoring. The critical care setting provides continuous access to intensivists, respiratory therapists, and nurses trained in NIV management and in interpreting physiological parameters critical to patient safety. Monitoring during helmet NIV involves an integrative assessment of multiple parameters and their trends over time, including oxygenation and carbon dioxide levels, respiratory rate, signs of respiratory muscle fatigue, dyspnea, patient–ventilator (a)synchrony via ventilator waveform analysis, and, in some cases, advanced techniques such as esophageal manometry [32,35,41–44]. Importantly, tidal volume cannot be reliably monitored with helmet NIV, as the large values displayed by the ventilator reflect both the gas entering the patient's respiratory system and the volume distending the interface; similarly, minute ventilation reflects system washout flow. Therefore, tidal volume should not be used as a predictor of treatment failure, as it is with facemask NIV [45].

The decision to escalate to invasive mechanical ventilation should be made promptly if helmet NIV fails to improve gas exchange, respiratory effort, or dyspnea. Delays in intubation have been associated with worse outcomes, underscoring the importance of ICU–based monitoring, where timely intervention is feasible [27].

The use of NIV for acute hypoxemic respiratory failure outside the critical care setting remains controversial. In step–down units or general wards, the absence of continuous monitoring and specialized personnel increases the risk of unrecognized deterioration. In emergency departments, helmet NIV has been explored primarily to provide early respiratory support in case of critical care unavailability. While early helmet NIV in this context may serve as a bridge to definitive care, it requires rapid assessment and transfer protocols to ensure safety.

In summary, intensive care units represent the optimal environment for helmet NIV due to the need for continuous, specialized monitoring, immediate recognition of treatment failure, and prompt escalation of care. Although its use in intermediate–care or emergency settings is possible [46], this requires appropriate infrastructure and trained staff to ensure both patient safety and treatment effectiveness.

## HELMET NONINVASIVE VENTILATION: HOW

Effective application of helmet NIV requires careful attention to ventilator settings to optimize patient-ventilator interaction and interface performance. Owing to the helmet's unique mechanical characteristics – including its large internal volume and compliance – standard settings used for facemask NIV often require substantial adjustment [15–17].

Higher PEEP levels, typically 10–14 cmH_2_O not only are clinically advantageous, but are also necessary to pressurize the interface and enhance performance. Similarly, moderately high pressure support (8–14 cmH_2_O) is preferred, with higher values recommended for patients exhibiting more intense inspiratory effort. When helmet CPAP is deemed the most appropriate strategy, the system must be set up utilizing high flow generators or Venturi systems ensuring adequate washout gas flow of at least 50 l/min, since the flow provided by mechanical ventilators in CPAP mode, instead, is not sufficiently high. Moreover, careful consideration should be given to the type of expiratory valves used, as they – together with set washout flow and HEPA filters – affect pressure regulation within the chamber and, consequently, the modulation of patient effort [47^▪^,^48^,^49^]. In addition, the pressurization time (i.e. the ramp) should be set as short as possible, to the lowest value permitted by the ventilator, as this is also crucial to improve synchronization and performance. If substantial leaks occur, helmet positioning should be adjusted or an alternative hood/collar size considered. When helmets equipped with anchoring armpit braces are used, care should be taken to avoid excessive pressure on the axillary region, which could lead to vascular complications [50,51]. A practical solution is to pull the braces downward and secure them to the patient's bed, keeping the helmet in place without increasing localized pressure. To minimize CO_2_ rebreathing, a double–limb circuit should be used instead of a Y-piece, with the two ports positioned on opposite sides [52].

To improve patient tolerance and allow prolonged, minimally interrupted treatments, light sedation may be employed. Dexmedetomidine is preferred because it minimally affects neural respiratory drive, making it a safer choice [53,54]. The use of remifentanil continuous infusion has also been reported [55]. However, extra attention should be paid while adopting sedation during NIV, as it may mask signs of treatment failure. Importantly, the overall effect of different sedation strategies during NIV on physiological and clinical outcomes has not yet been systematically explored [56,57].

Finally, a double–limb circuit without humidification is generally optimal for patients’ comfort during helmet NIV, as adequate conditioning and humidification of inspired gas are naturally provided by breathing within a closed air reservoir [58].

## CONCLUSION

Helmet NIV combined with careful monitoring in specialized environments offers a valuable tool in the noninvasive management of moderate-to-severe acute hypoxemic respiratory failure (PaO_2_/FiO_2_ < 200 mmHg), particularly when applied early, for prolonged periods, and with individualized settings based on inspiratory effort and clinical status. Interface-related peculiarities characterizing patient-ventilator interaction may represent key differences with facemask NIV in terms of effort modulation and insufflation patterns for P-SILI prevention. With growing physiological evidence, ongoing trials will better define its role alongside other noninvasive respiratory support strategies.

## Acknowledgements

*AI disclosure: The authors disclose the use of ChatGPT (OpenAI, San Francisco, CA) for language and grammar editing; all authors reviewed and approved the final text and are responsible for its content.*

### Financial support and sponsorship

*None.*

### Conflicts of interest

*M.A. has received payments for Board participation from Maquet, Air Liquide and Chiesi, and a research grant by GE. D.L.G. has received payments for travel expenses by Getinge, Draeger and Hamilton, personal fees by Draeger, and research grants by Fisher and Paykel and GE.*

## REFERENCES AND RECOMMENDED READING

Papers of particular interest, published within the annual period of review, have been highlighted as:

▪ of special interest
▪▪ of outstanding interest
